# Primary Ewing’s Sarcoma of the Penis: First Reported Case in the United Kingdom

**DOI:** 10.7759/cureus.31698

**Published:** 2022-11-20

**Authors:** Peter Estaphanous, David Dickerson, Francesca Maggiani, Alireza Vosough, Aditya Manjunath

**Affiliations:** 1 Urology, Bristol Urological Institute North Bristol NHS Trust, Bristol, GBR; 2 Pathology, North Bristol NHS Trust, Bristol, GBR; 3 Radiology, North Bristol NHS Trust, Bristol, GBR

**Keywords:** urologic cancer, uro oncology, ewing's sarcoma, penile cancer, urology, andrology, penile tumour, extraskeletal ewing's sarcoma, sarcoma penis, extraosseous ewing's sarcoma

## Abstract

Penile cancer is generally rare, and Squamous cell cancer of the penis is the most common histological type. Sarcoma of the penis has a low incidence, but they tend to grow faster than other penile cancers. One of the rarest types of penile sarcomas is Extra-Skeletal Ewing's Sarcoma (EES). The management of such cases can be challenging, and treatment guidelines do not exist for these rare cases. We present a rare case of EES that has developed in the penis of a young patient in the United Kingdom.

## Introduction

Ewing's sarcoma (ES) is a rare tumor; however, it is considered the second most common bone tumor of malignant nature in children [1]. It falls among a family of tumors called Ewing's sarcoma family, with primitive neuroectodermal tumors (PNET) and Askin tumors [2]. ES commonly arises from the bone marrow cavity of the pelvis, long bones, and ribs [3]. It is known to be an aggressive and poorly differentiated tumor with a high potential to recur and metastasize due to its increased biological activity [4,5]. The spread is commonly hematogenous and rarely lymphatic. Given that ES is a chemo-sensitive and radio-sensitive tumor, the principles of ES treatment consist of combined neoadjuvant chemotherapy followed by local treatment with surgery. Radiotherapy is also considered but prefers surgical treatment over irradiation alone [6].

If ES primarily arises from soft tissue, it is called Extra-skeletal Ewing's sarcoma (EES). It represents 10-20% of ES, commonly arising from extremities, chest wall, paravertebral space, and retroperitoneum. Rare locations were also reported, such as the heart, kidneys, jejunum, larynx, and vulva [7]. Primary EES of the Penis is extremely rare, and less than 10 cases are reported worldwide [5]. We present the first case to be registered in the United Kingdom.

## Case presentation

An 18-year-old male patient, who does not have any significant past medical or family history, presented to his local hospital with a progressively enlarging painless lump at the base of the penis on the left side over three months. It has not affected his urinary or erectile function, and he did not report any other systemic symptoms such as weight loss or loss of appetite.

Clinical examination revealed a 6 cm firm mass at the penoscrotal junction, which felt attached to the underlying penile structures. Both testicles were spared and of normal size and consistency, and there was no palpable regional lymphadenopathy.

**Figure 1:**
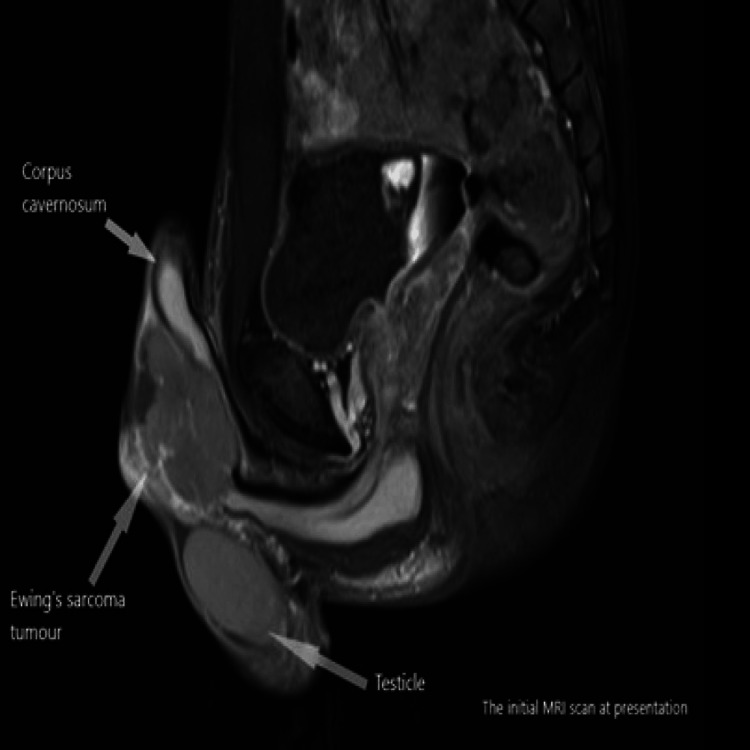
Initial Magnetic Resonance Imaging (MRI) scan at presentation (sagittal plane).

**Figure 2:**
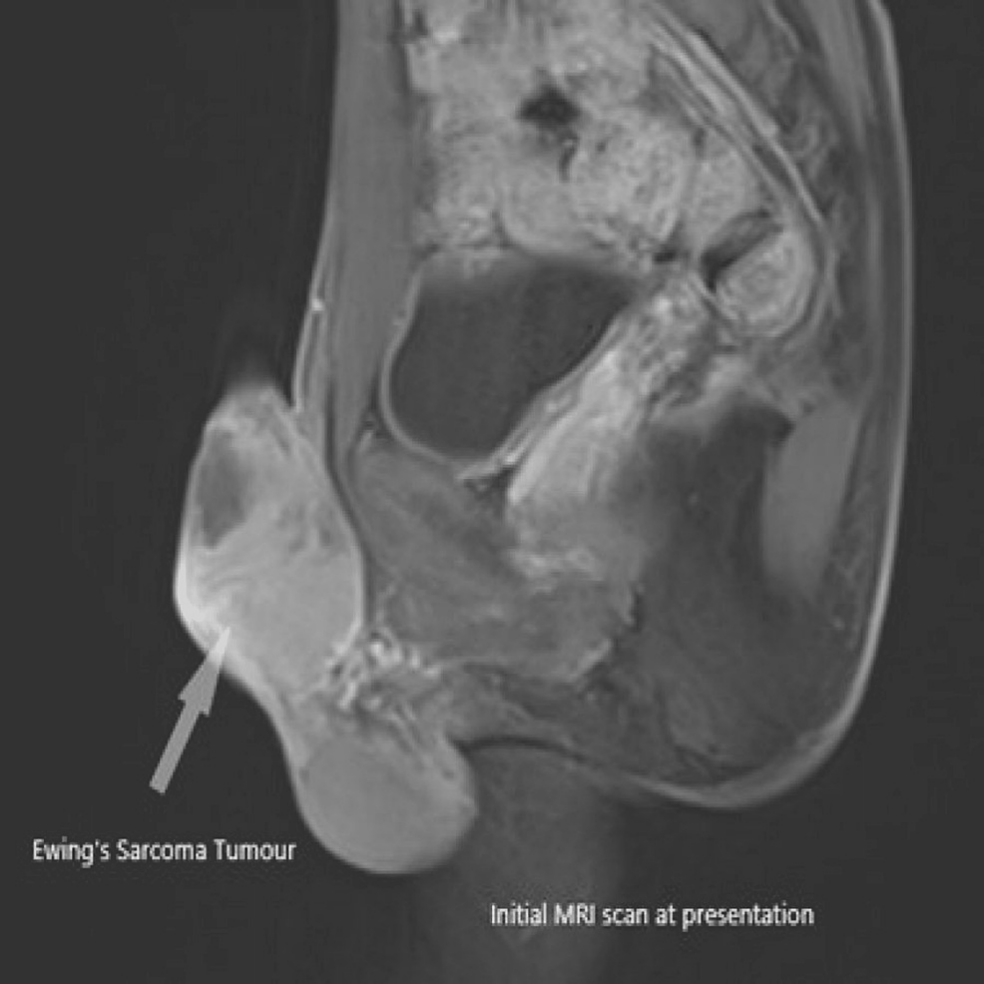
Initial MRI scan at presentation (sagittal plane).

**Figure 3:**
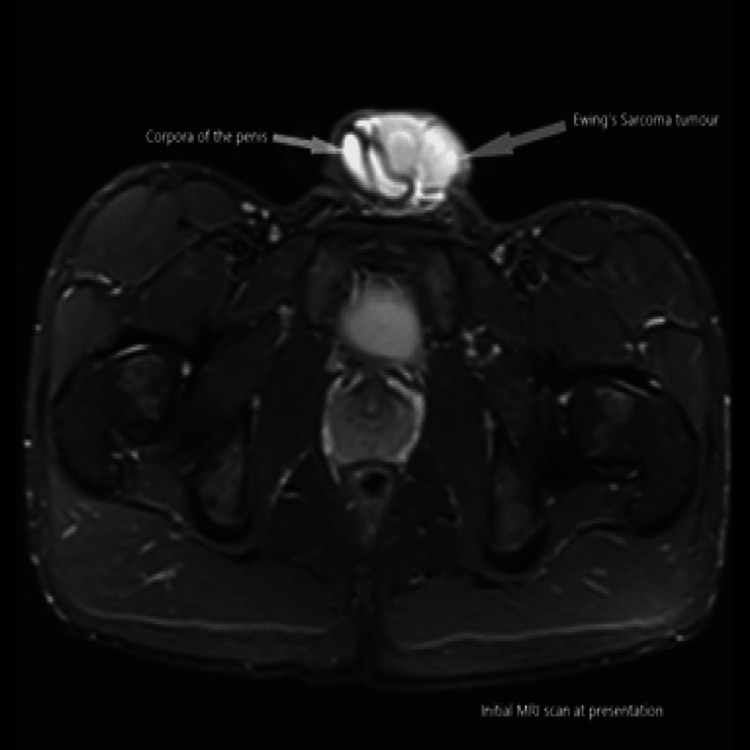
Initial MRI scan at presentation (axial plane)

Magnetic Resonance Imaging (MRI) of the penis has been arranged. It showed a 5.5 cm enhancing mass at the base of the penis that appeared separate from the underlying corpora cavernosa (Figures 1-3). Staging Computed Tomography (CT) scan confirmed no evidence of metastatic disease.

An incision biopsy was performed. Immunohistochemistry revealed tumor cells that showed positivity with CD99, focal positivity with CD56, and negativity with CD45, CD20, CD3, S100, myogenin, WT1, MYO-D1, and myeloperoxidase. Scattered cells also express Epithelial Membrane Antigen (EMA). The features of a poorly differentiated round cell sarcoma were consistent with a soft tissue Ewing's sarcoma. This diagnosis has been confirmed by demonstrating a characteristic EWSR1 (22q12.2) rearrangement detected by Fluorescence In Situ Hybridisation (FISH). 

Given the rarity of the case, the histopathological diagnosis was challenging. The case was sent for an expert pathologist review in a highly specialized orthopedic center, which agreed with the diagnosis of EES. 

A staging fluorodeoxyglucose (FDG) Positron Emission Tomography-Computed Tomography (PET/CT) scan followed and confirmed FDG avid lesion of the penis in keeping with the known sarcoma. No FDG avid metastatic disease was demonstrated; however, some mildly prominent retroperitoneal lymph nodes were believed to be reactive rather than malignant.

**Figure 4:**
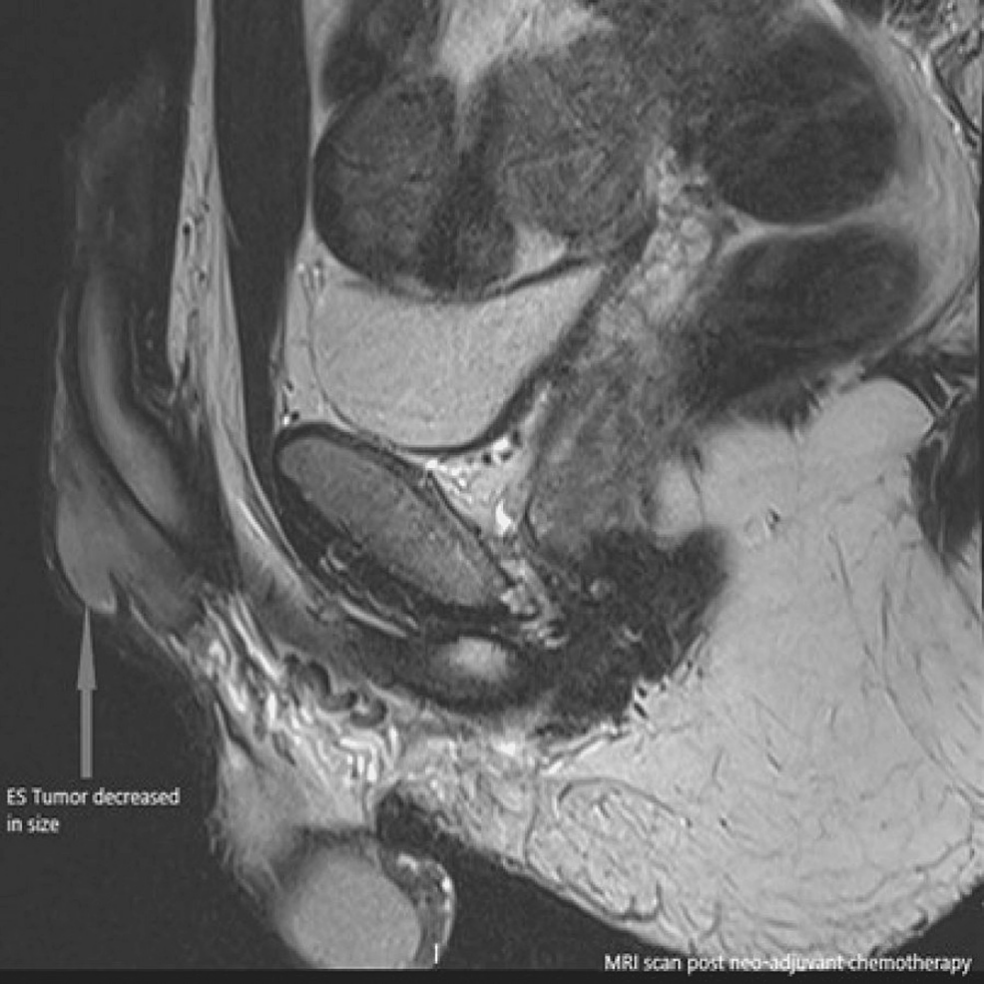
MRI scan post-neo-adjuvant chemotherapy showing interval decrease of the tumor size (sagittal plane).

**Figure 5:**
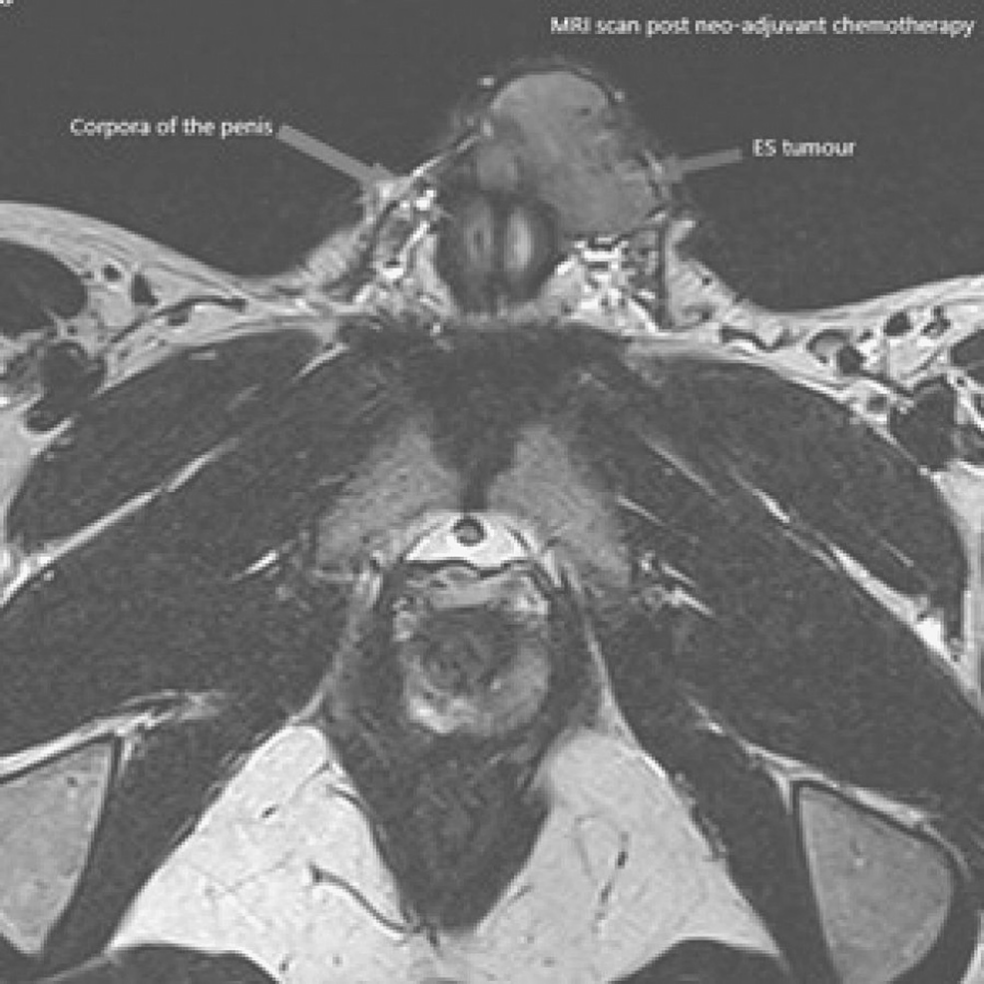
MRI scan post-neo-adjuvant chemotherapy showing interval decrease of the tumor size (axial plane).

**Figure 6:**
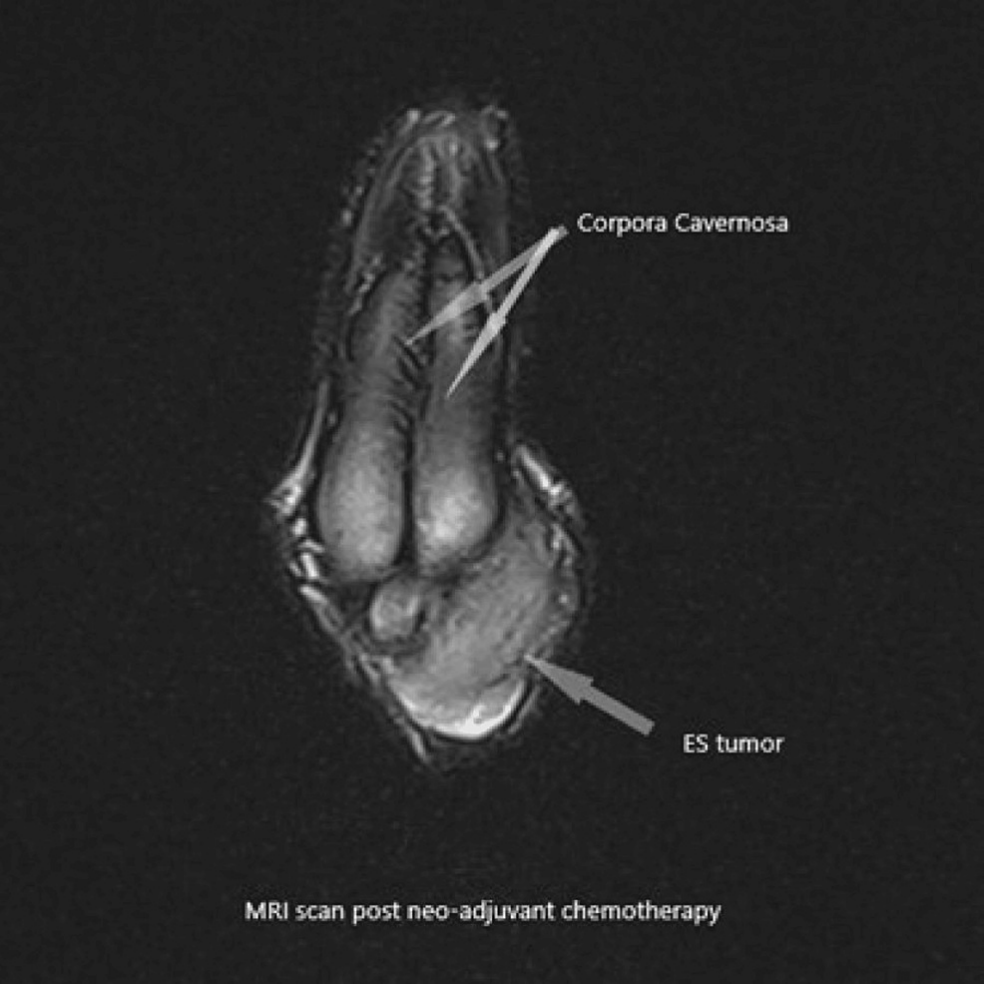
MRI scan post-neo-adjuvant chemotherapy showing interval decrease of the tumor size (coronal plane).

The case was reviewed in the Supra-Regional Penile Cancer multi-disciplinary team meeting (MDT), which questioned the involvement of the urethra as it was not clear on the initial MRI scan. The imaging review was suspicious for tumor extension beyond the pubic symphysis. According to Ewing's sarcoma protocol, the patient received neo-adjuvant chemotherapy, which involved 11 cycles of alternating Vincristine/Doxorubicin/Cyclophosphamide and Ifosfamide/Etoposide. He was also offered sperm storage. He had an excellent response to the neo-adjuvant chemotherapy with significant downsizing of the tumor seen on serial imaging (Figures 4-6).

The patient then underwent radical total penectomy and perineal urethrostomy, which included an elliptical incision around the base of the penis, mobilization of the penis 4cm proximal to the mass, and routine hemisection of the penis and the urethra 1.5cm proximal to the mass than the creation of a perineal urethrostomy. Leaving an adequate stump for consideration of surgical reconstruction while ensuring adequate excision with clear margins is the challenge of the operation.

**Figure 7:**
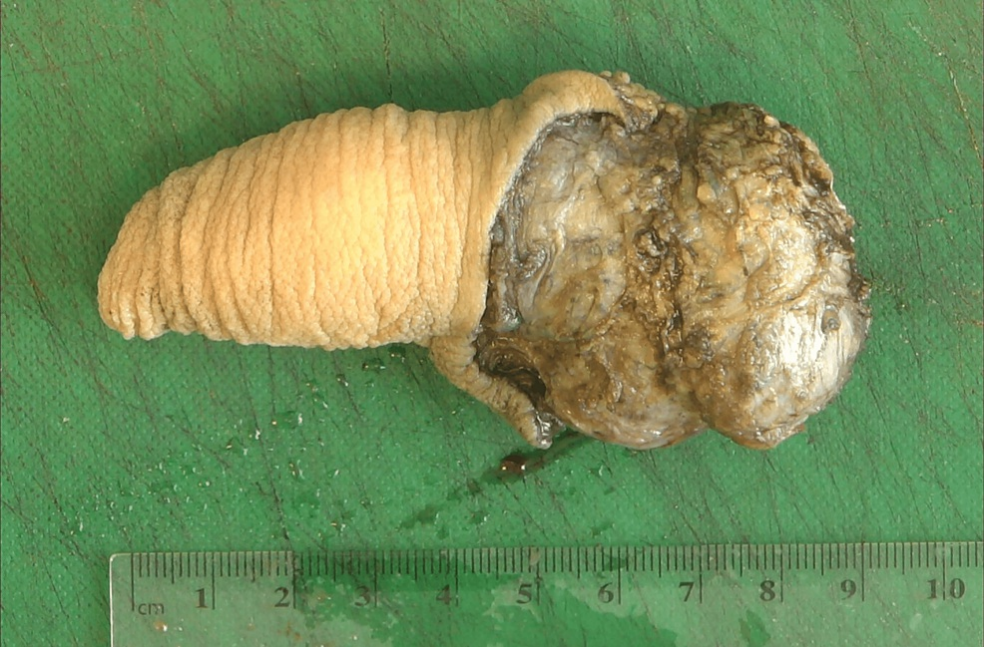
Pathological specimen showing a dorsal view of the penis and the tumor.

**Figure 8:**
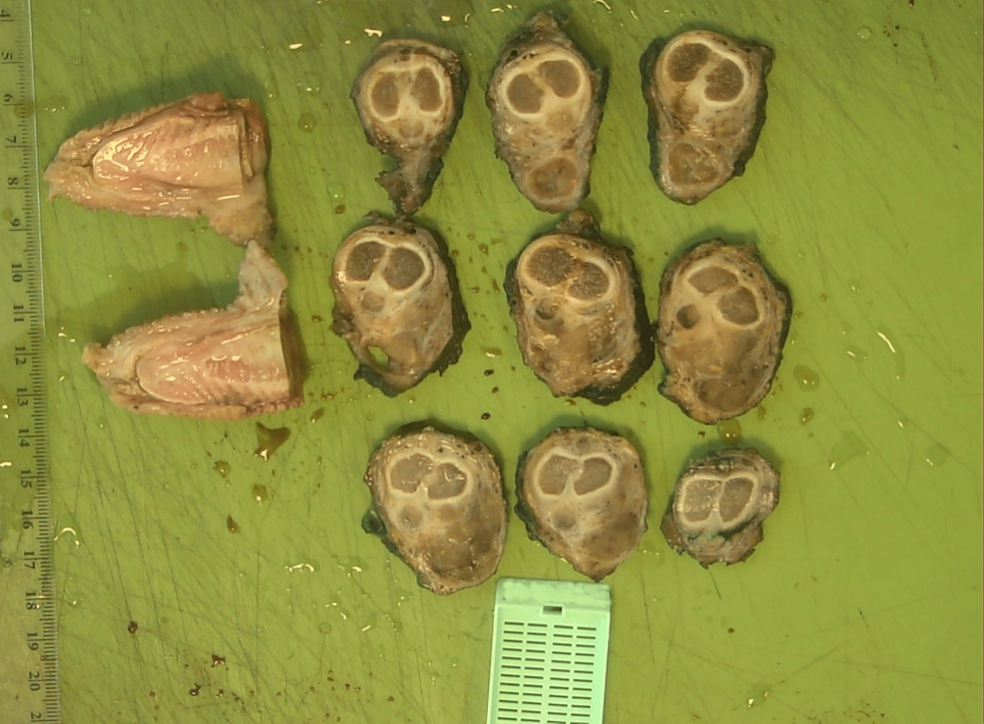
Pathological specimen showing sections through the penis and the tumor.

**Figure 9:**
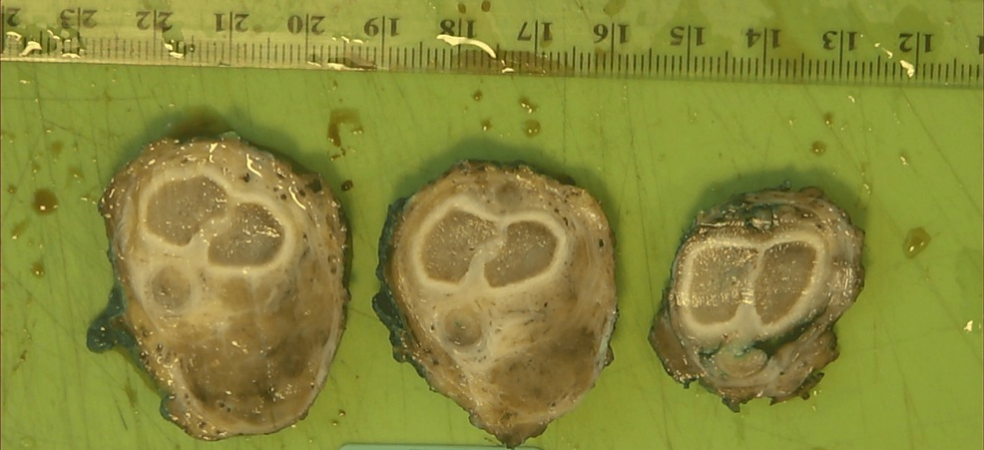
Pathological specimen showing axial sections through the penis and the tumor.

**Figure 10:**
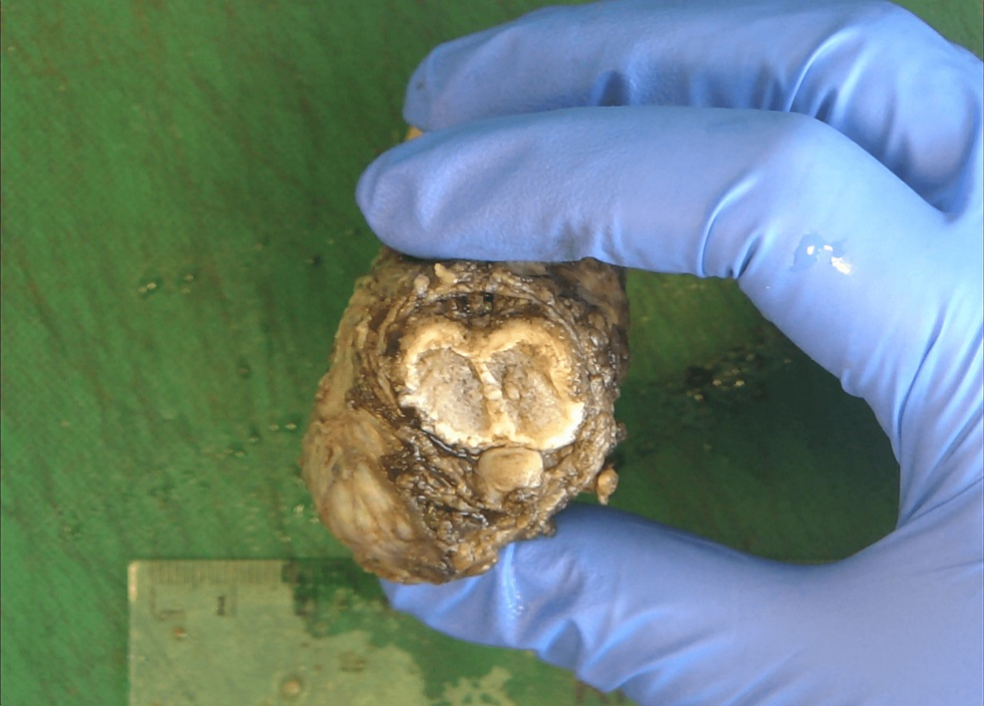
Pathological specimen showing axial sections through the penis and the tumor.

The specimen was sent for histopathological assessment (Figures 7-10). Postoperatively, he recovered well and received psychological support from Psychological Health Services and the Teenage and Young Adult Cancer Service (TYA). As per ES protocol, the patient received three more chemotherapy cycles of alternating Vincristine/Doxorubicin/Cyclophosphamide and Ifosfamide/Etoposide. After a period of cancer-free survival, the patient will be considered for total phallic reconstruction if he wishes.

Histopathology confirmed that the morphology and immunophenotype are compatible with the original diagnosis of Ewing's sarcoma, with chemotherapy-induced fibrotic changes of the adjacent stroma. The surgical margins were confirmed to be clear. According to the protocol for follow-up of intermediate and high-grade soft tissue sarcoma, the patient will have three monthly follow-ups for one year, four monthly follow-ups until year 3, 6 monthly follow-ups until year five, then yearly follow-ups for 10 years, and then discharge can be considered. The follow-ups might include regular MRI or CT scans and chest X-rays depending on the patient's symptoms and the judgment of the oncologist or the surgeon.

## Discussion

Extraskeletal occurrence of ES is rare; however, it can occur in any location [8]. Upon review of the medical literature, we identified cases reported in female and male genital organs such as the vulva, vagina, uterus, ovaries, and testicles [9,10]. Even so, the occurrence of ES in the penis is infrequent. Six cases of primary ES of the penis have been reported. Two cases in China [7,11], one in India [12], one in the United States of America (USA) [13], one in Turkey [14], and one in the Czech Republic [5]. The youngest of these cases was 12 years old, and the oldest was 32 years old [5,11].

**Figure 11:**
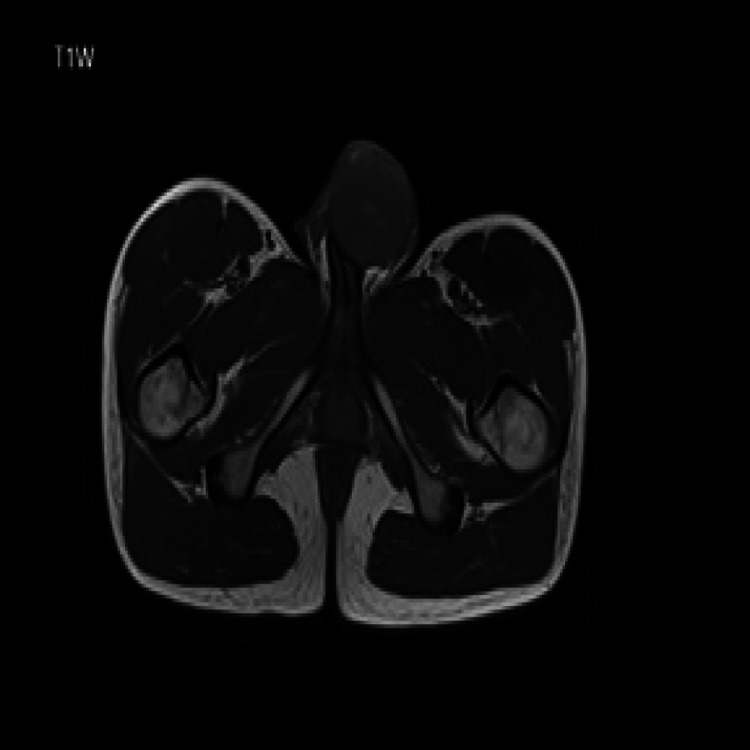
T1W image

**Figure 12:**
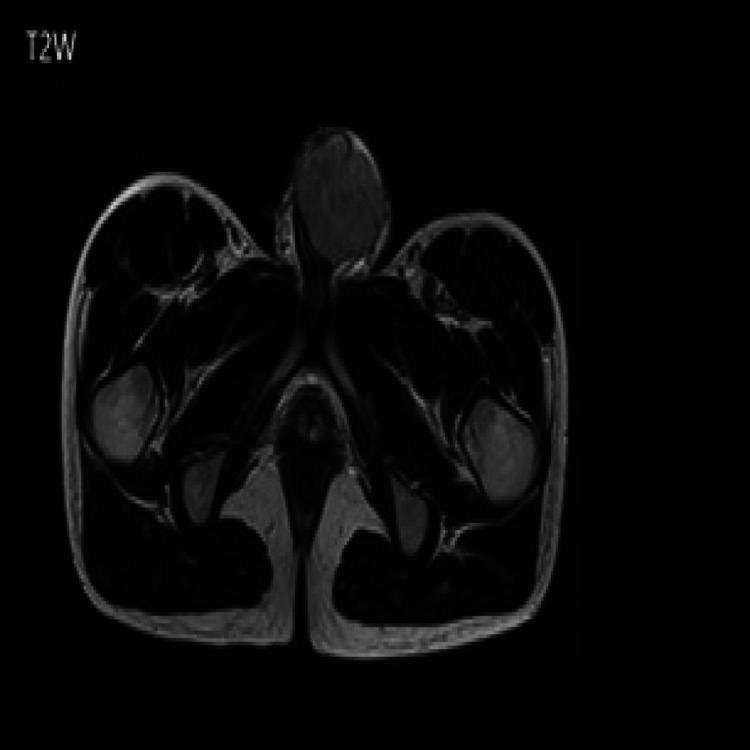
T2W image

The diagnosis is made with imaging, and a biopsy obtains a tissue diagnosis. MRI scan with prostaglandin injection to artificially induce erection is the imaging modality of choice for penile lesions [12]. MRI features include iso-intensity on T1-weighted (T1W) (Figure 11) and hypo-intensity on T2-weighted (T2W) (Figure 12).

Histologically, EES is composed of round or oval cells with round hyperchromatic nuclei separated by fibrous septa. These cells are usually rich in glycogen [15]. Immunohistochemically, EES shows positivity for CD99, CD56, and less commonly to Neuron Specific Enolase (NSE) and S-100. The FISH test is also helpful in establishing the presence of the t11:12 translocation [16].

EES is chemo and radiosensitive. Treatment is, therefore, multi-modal, neo-adjuvant chemotherapy and surgical treatment with or without adjuvant radiotherapy [6]. Psychological support is crucial, especially for young patients, and it helps with tolerating chemotherapy's debilitating side effects and in coming up with the functional and psychological effects of penile amputation. If feasible, future reconstruction should be considered, especially in a young patient, and it should be included in discussion with the patient. Surgical resection can factor in future reconstruction by leaving a small penile remnant and maintaining urethral length to facilitate phalloplasty.

## Conclusions

The occurrence of primary penile EES is infrequent. Our case exemplifies how a multi-disciplinary approach in a specialized penile cancer center is essential in providing optimum oncological, functional, and psychological care and outcomes in such rare cases. As it is a rare case, no guidelines for management are available. Our case highlights the required investigations for diagnosis and the steps of management. It also emphasizes the role of psychological support, especially for young patients, and the importance of taking future reconstruction into account.
